# Consequences of oncogenic isocitrate dehydrogenase mutations and 2-hydroxyglutarate production in prostate epithelium

**DOI:** 10.1186/1756-8935-6-S1-P120

**Published:** 2013-04-08

**Authors:** Jon A Over, Jonathan D Licht

**Affiliations:** 1 Department of Hematology/Oncology, Northwestern University Feinberg School of Medicine, Chicago, 60611, USA

## Background

Recent technological advances in DNA sequencing have yielded more comprehensive identification of mutations occurring in tumors. These efforts have detected recurrent mutations in a gene encoding isocitrate dehydrogenase (IDH1) in several tumor types including prostate cancer. In each of these tumors IDH1 missense mutations at arginine 132 (R132) confer a gain of function and produce an aberrant metabolite, 2-hydroxyglutarate (2HG) as opposed to wild type (WT) activity that produces alpha-ketoglutarate (a-KG). a-KG is a cofactor for the Ten-Eleven Translocation enzymes (TET1/2/3), which catalyze DNA hydroxymethylation. Although this DNA modification is predicted to affect epigenetic regulation, its specific effects and target genes remain unclear. We hypothesize that 2HG produced by mutant IDH1 promotes tumor phenotypes through mechanisms directly affecting DNA hydroxymethylation.

## Materials and methods

We tested our hypothesis in a cell culture model using RWPE cells, which were derived from a histologically normal adult human prostate. These cells were infected with retrovirus co-expressing green fluorescent protein together with either WT or R132H alleles of IDH1. 2HG levels were quantified by a fluorometric assay that measures NADH produced by 2HG-dehydrogenase. DNA hydroxymethylation was measured in genomic DNA samples by dot blots with immunodetection using a rabbit polyclonal antibody (Active Motif #39770). Gene expression was measured by quantitative-PCR on cDNA prepared from total RNA isolations. Cell proliferation was evaluated by measuring cellular conversion of methylthiazolyldiphenyltetrazolium (MTT) to purple-colored formazan that is quantified by measuring absorbance at λ = 600 nm.

## Results

RWPE cells expressing the R132H mutant produced 2HG at levels comparable to those measured in cancer cell lines with endogenous IDH1 mutations, whereas 2HG was not generated by IDH1 WT overexpression. Relative to WT-expressing or uninfected cells we observed that global DNA hydroxymethylation was reduced in R132H-expressing cells. Moreover, our data suggest that this trend increases with duration of R132H expression. Reduced DNA hydroxymethylation likely results from inhibition of TET2 and/or TET3, because TET1 expression was not detected in RWPE cells. Despite molecular changes associated with IDH1 R132H expression, proliferation was not affected when cells were cultured in complete media. However, 2HG conferred a growth advantage to RWPE cells cultured with reduced growth factor levels.

## Conclusions

Production of 2HG by mutant IDH1 expression was sufficient to reduce global levels of DNA hydroxymethylation in prostate epithelial cells. Moreover, these cells exhibited increased proliferation upon 2HG exposure when cultured in media with diminished growth factors, which is intended to better mimic resource limitations of tumor growth. Collectively these findings improve our understanding of how IDH1 mutations promote carcinogenesis with the goal of developing effective targeted therapies against IDH1 mutant tumors.

